# A new graph-based method for pairwise global network alignment

**DOI:** 10.1186/1471-2105-10-S1-S59

**Published:** 2009-01-30

**Authors:** Gunnar W Klau

**Affiliations:** 1 CWI, P.O. Box 94079, 1090 GB Amsterdam, The Netherlands

## Abstract

**Background:**

In addition to component-based comparative approaches, *network alignments *provide the means to study conserved network topology such as common pathways and more complex network motifs. Yet, unlike in classical sequence alignment, the comparison of networks becomes computationally more challenging, as most meaningful assumptions instantly lead to *NP*-hard problems. Most previous algorithmic work on network alignments is heuristic in nature.

**Results:**

We introduce the graph-based *maximum structural matching *formulation for pairwise global network alignment. We relate the formulation to previous work and prove *NP*-hardness of the problem.

Based on the new formulation we build upon recent results in computational structural biology and present a novel Lagrangian relaxation approach that, in combination with a branch-and-bound method, computes provably optimal network alignments. The Lagrangian algorithm alone is a powerful heuristic method, which produces solutions that are often near-optimal and – unlike those computed by pure heuristics – come with a quality guarantee.

**Conclusion:**

Computational experiments on the alignment of protein-protein interaction networks and on the classification of metabolic subnetworks demonstrate that the new method is reasonably fast and has advantages over pure heuristics. Our software tool is freely available as part of the LISA library.

## Background

In systems biology, complex biological systems are often modeled as networks. Examples include protein-protein interaction (PPI), metabolic, gene-regulatory, and signal transduction networks. The increasing quality and quantity of available data creates the need for automated analysis methods to better understand cellular processes, network organization, evolutionary changes, and disease mechanisms [1,2]. Based on the assumption that evolutionary conservation implies functional significance, comparative approaches may help improve the accuracy of data, elucidate protein pathways and complexes, generate, investigate, and validate hypotheses about the underlying networks, and transfer functional annotations. In addition to component-based comparative approaches, *network alignments *provide the means to study conserved network topology such as common pathways and more complex network motifs. Yet, unlike in classical sequence alignment, the comparison of networks becomes computationally more challenging, as most meaningful assumptions instantly lead to *NP*-hard problems.

### Previous work

One of the first contributions to automatic biological network alignment is [3], where the authors introduce a concept later called *global alignment graph *and find functionally related enzyme clusters in metabolic networks using a simple heuristic. Kelley *et al*. [4] formalize the concept and present the PATHBLAST algorithm, which heuristically finds high-scoring common paths in two protein-protein interaction networks using randomized dynamic programming. Detecting more complex shared topologies has been addressed by Sharan *et al*. [5], where the authors introduce a probabilistic model for protein complexes and propose a heuristic greedy approach to search for dense subgraphs in the global alignment graph, which correspond to significant shared complexes in the original PPI networks. Koyutürk *et al*. [6] also use the global alignment graph with a more elaborate scoring scheme to compute pairwise alignments of PPI networks. Narayanan and Karp [7] compare two PPI networks using a different model based on a graph-matching algorithm. They restrict the structural conservation to the environment of a node and thus achieve a polynomial running time.

While most of the above approaches aim at computing local alignments, a recent method by Singh *et al*. [8] focuses explicitly on computing global alignments between protein interaction networks. They heuristically approach the problem by preferably matching nodes which have a similar neighborhood, which they encode as an eigenvalue problem.

For multiple network alignment, the method from [5] has been adapted in [9]. Koyutürk *et al*. [10] determine multiple alignments by contracting the global alignment graph and then applying algorithms from frequent itemset extraction. Jaeger and Leser [11] determine conserved subgraphs among *k *PPI networks using a heuristic for multidimensional matching in a *k*-partite graph that results from linking each protein to its best ortholog match candidate in each of the other networks. The GRAEMLIN algorithm [12] uses local search to construct a global multiple alignment. Singh *et al*. have adapted their method for the multiple case [13].

### Contribution

In this paper, we introduce the *maximum structural matching *formulation for global network alignment and show its relation to the *global alignment graph*. We derive integer linear programming formulations for the maximum structural matching problem and a Lagrangian relaxation algorithm based on these formulations. To our knowledge, this is the first contribution to the relatively young field of biological network alignment that does not approach the problem heuristically. Still, our computational results indicate that the Lagrangian approach is reasonably fast to provably optimally align even large networks. We present preliminary results from two ongoing proof-of-concept studies, where we use the method to globally align protein-protein-interaction networks and to classify metabolic subnetworks.

Note that this is a methodological paper whose purpose is to introduce the new approach with mathematical rigor. The two proof-of-concept studies demonstrate the potential of the method in practice. However, a detailed comparison to other methods is beyond the scope of this article and will be carried out as future work.

## Methods

### A combinatorial formulation for network alignment

In this section we give a formal definition of network alignment. We define the global pairwise network alignment problem and present a graph-theoretical reformulation, which is an extension of the *maximum weight trace *formulation, which has been proposed by Kececioglu for classical sequence alignment [14]. Furthermore, we relate our definition to previous work.

In analogy to the classical sequence case, we define a pairwise alignment of two networks as follows. Note that this definition is already quite close to the formulation presented later in this section and can readily be extended to multiple network alignment. Let "-" denote the gap symbol.

**Definition 1 **(Network alignment). *Given two networks G*_1 _= (*V*_1_, *E*_1_) *and G*_2 _= (*V*_2_, *E*_2_), *a *network alignment *a*: *V*_1 _→ *V*_2 _∪ {-} *maps a vertex i *∈ *V*_1 _to

$$a(i)=\{\begin{array}{ll} j\in V_{2} & a\;vertex\;j\;in\;the\;second\;network\\ - & a\;gap. \end{array}$$

Note that in contrast to sequence alignments, network alignments do not have to respect an inherent sequential order of the objects to align.

**Definition 2 **(Score). *The score of a network alignment a*: *V*_1 _→ *V*_2 _∪ {-} *of two networks G*_1 _= (*V*_1_, *E*_1_) *and G*_2 _= (*V*_2_, *E*_2_) *is*

$$s(a)=\sum_{\begin{array}{c} i\in V_{1}\\ a(i)\neq - \end{array}}\sigma (i,a(i))+\sum_{\begin{array}{c} i\in V_{1}\\ a(i)\neq - \end{array}}\sum_{\begin{array}{c} k\in V_{1}\\ a(k)\neq - \end{array}}\tau (i,a(i),k,a(k)),$$

where *σ*: *V*_1 _× *V*_2 _→ ℝ^≥0 ^*gives the score of mapping individual nodes onto each other and τ*: *V*_1 _× *V*_2 _× *V*_1 _× *V*_2 _→ ℝ^≥0 ^*gives the score of mapping pairs of nodes onto each other*.

**Figure 1:**
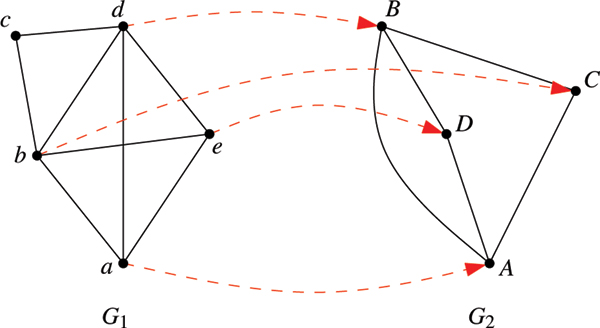
**Network alignment *a***. A dashed arrow from a node *i *∈ *V*_1 _from the first network *G*_1 _= (*V*_1_, *E*_1_) to a node *j *∈ *V*_2 _from the second network *G*_2 _= (*V*_2_, *E*_2_) indicates that *a*(*i*) = *j*. Unmapped vertices are mapped to gaps. The score of the alignment depends on the values given in *σ *and *τ*. For simplicity, we assume that *σ*(*i*, *j*) = 1 for all *i *∈ *V*_1 _and *j *∈ *V*_2 _and that *τ*(*i*, *j*, *k*, *l*) = 1 if (*i*, *k*) ∈ *E*_1 _and (*j*, *l*) ∈ *E*_2 _and *τ*(*i*, *j*, *k*, *l*) = 0 otherwise. This leads to a score of 4 + 5 = 9 in the example.

This definition allows a quite flexible modeling of scores, which may be used to express mismatches and gaps, and which can also be based on additional information, such as, for example, edge weights. Typically, the *σ*-part of the scoring function will be based on pairwise similarity of the objects represented by the nodes and will assign, say, similar proteins in two protein-protein interaction networks a high score, whereas the *τ*-part will reward conserved interactions between pairs of nodes. Note that the definition is similar to structural alignment scoring functions as, for example, used to compare RNA molecules [15]. Figure 1 illustrates the definitions.

Given these definitions, we are able to define the network alignment problem formally:

**Definition 3 **(Pairwise global network alignment). *Given two networks G*_1 _= (*V*_1_, *E*_1_) *and G*_2 _= (*V*_2_, *E*_2_) *and a scoring function s as defined in Def. 2, the *pairwise global network alignment problem asks for a highest-scoring alignment A* *of G*_1 _*and G*_2_, *that is*, $A^{*}=\arg\max_{A\in A}s(A)$*s*(*A*), *where *A*denotes the set of all possible alignments of G*_1 _*and G*_2_.

**Theorem 1**. *The pairwise global network alignment problem as defined in Def. 3 is NP-hard*.

*Proof*. It is easy to see that the pairwise network alignment problem is in *NP*, since a non-deterministic algorithm needs only guess the best alignment *a*. We prove *NP*-hardness by a simple reduction from the maximum common subgraph problem. A common subgraph of two graphs *G*_1 _= (*V*_1_, *E*_1_) and *G*_2 _= (*V*_2_, *E*_2_) is characterized by subsets *E*_1_' ⊆ *E*_1 _and *E*_2_' ⊆ *E*_2 _such that the two subgraphs ${G^{\prime }}_{1}$ = (*V*_1_', *E*_1_') and ${G^{\prime }}_{2}$ = (*V*_2_', *E*_2_') are isomorphic, where *V*_1_' and *V*_2_' denote the vertices that are the endpoints of edges in *E*_1_' and *E*_2_', respectively. A maximum common subgraph is a common subgraph with the maximum number of edges, and its computation is a well-known *NP*-hard problem [16].

We can solve the maximum subgraph problem with an algorithm for network alignment by simply using the following scoring function:

*σ*(*i*, *j*) = 0 for all *i *∈ *V*_1_, *j *∈ *V*_2_

and

$$\tau (i,j,k,l)=\{\begin{array}{ll} 1 & \begin{array}{ccc} \text{for }(i,k)\in E_{1} & \text{and} & (j,l)\in E_{2} \end{array}\\ 0 & \text{otherwise}. \end{array}$$

A best network alignment will then correspond to a maximum common subgraph.   □

The above definition of network alignment is very close to the notion of *trace *as introduced by Kececioglu for classical sequence alignment [14]. We give an analogous definition for the alignment of networks:

**Definition 4 **(Alignment graph). *Given two networks G*_1 _= (*V*_1_, *E*_1_) *and G*_2 _= (*V*_2_, *E*_2_), *the *alignment graph *A is a complete bipartite edge-weighted graph with vertex set V*_1 _∪ *V*_2_. *The weight of an edge e *= (*i*, *j*) *with i *∈ *V*_1 _*and j *∈ *V*_2 _*is w*(*e*) = *σ*(*i*, *j*) *and represents the gain of aligning the endpoints of the edge*.

**Figure 2:**
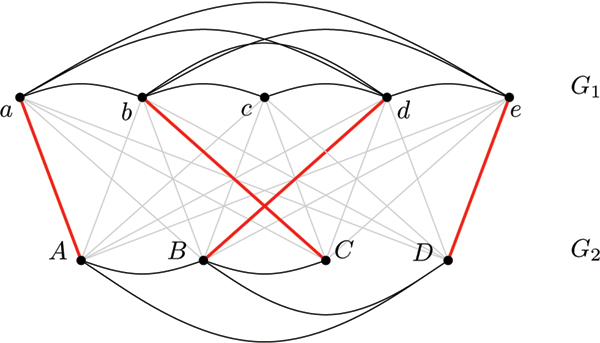
**Alignment graph**. Alignment graph *A *for the alignment *a *from Fig. 1. Heavier alignment edges are realized by *a*.

Figure 2 shows the alignment graph for the instance given in Fig. 1. In analogy to the sequence case, we say that a network alignment *a realizes *an edge (*i*, *j*) in *A *if *a*(*i*) = *j*. Similar to the trace formulation we strive to establish a connection between an alignment *a *and the alignment graph *A*. As the order of the vertices does not play a role, this connection is precisely characterized by the graph-theoretical concept of *matching*. A matching in a graph is a subset of its edges such that no two chosen edges share a common endpoint.

**Observation 1**. *There is a one-to-one correspondence between matchings in the alignment graph and network alignments*.

The alignment graph provides an alternative way to represent an alignment of the nodes in a network. Yet, in the basic version we are unable to deal with structural conservation. Therefore we introduce the concept of *structural matches*, which have already been used for structural alignment, where they are referred to as *interaction matches *[15].

**Definition 5 **(Structural match). *A *structural match *is a pair of alignment edges *(*i*, *j*), (*k*, *l*) *in the alignment graph. We say that a network alignment *realizes *a match *(*i*, *j*), (*k*, *l*) *if it realizes both alignment edges *(*i*, *j*) *and *(*k*, *l*).

We are now able to reformulate the pairwise global network alignment problem as a combinatorial problem in the alignment graph. Let > denote an arbitrary order of the edges in *A*.

**Definition 6 **(Maximum structural matching). *Given two networks G*_1 _= (*V*_1_, *E*_1_) *and G*_2 _= (*V*_2_, *E*_2_) *and a scoring function s, the structural score of a matching M in the alignment graph A is given by*

$$s(M)=\sum_{(i,j)\in M}\sigma (i,j)=\sum_{(i,j)\in M}\sum_{\begin{array}{c} (k,l)\in M\\ \left(k,l)>(i,j\right) \end{array}}\tau (i,j,k,l).$$

*The *maximum structural matching problem *asks for a highest-scoring structural matching*.

Observation 1 straightforwardly extends to the structural case and yields the following result.

**Lemma 1. ***Consider a network alignment a and the matching M it realizes in the alignment graph. Then we have s*(*a*) = *s*(*M*).

This allows us to concentrate on the alignment graph to find the best pairwise global network alignment. In the next section, we present an integer linear programming approach to determine a maximum structural matching in a bipartite graph.

Note that our definition of alignment graph is different, but in a sense equivalent, to the *global alignment graph *concept used in the PATHBLAST algorithm [4] and first introduced in [3]. The following observation relates the two concepts for the case of pairwise alignment; the multiple case is analogous. The global alignment graph contains weighted nodes for pairs of nodes in the original networks – which correspond to the alignment edges in our bipartite alignment graph – and weighted edges represent conserved interactions, gaps, or mismatches – which correspond to structural matches in our definition. Weights of nodes and edges correspond to the weights of alignment edges and structural matches, respectively. Determining clique-like heavy subgraphs in the global alignment graph – for which several heuristics have been presented – is equivalent to our definition of network alignment as a maximum structural matching in our alignment graph. We nevertheless prefer our alternative definition, because it allows us to employ the well-studied field of matchings in bipartite graphs as the next sections will show.

### Integer linear programming formulations for network alignment

We can straightforwardly cast the maximum structural matching problem as a non-linear integer program.

For each edge (*i*, *j*) ∈ *V*_1 _× *V*_2 _of the alignment graph, we define a binary variable *x*_*ij *_with the interpretation *x*_*ij *_= 1 if (*i*, *j*) is part of the structural matching and *x*_*ij *_= 0 otherwise. Let *δ*(*v*) denote the set of edges incident to vertex *v*. The formulation is then

(1) $$\max\sum_{(i,j)\in V_{1}\times V_{2}}\sigma (i,j)x_{ij}+\sum_{(i,j)\in V_{1}\times V_{2}}\sum_{(k,l)\in V_{1}\times V_{2}}\tau (i,j,k,l)x_{ij}x_{kl}$$

(2) $$\begin{array}{cc} \text{s}.\text{t}.\sum_{\left(i,j)\in \delta (v\right)}x_{ij}\leq 1 & \forall v\in V_{1}\cup V_{2} \end{array}$$

(3) *x*_*ij *_∈ {0, 1}   ∀(*i*, *j*) ∈ *V*_1 _× *V*_2_

Inequalities (2) make sure that the choice of alignment edges corresponds to a matching in the bipartite graph and go back to Birkhoff's theorem [17]. Linearization leads to the following *integer linear program *(ILP), which forms the basis of our Lagrangian relaxation approach. We define variables *y*_*ijkl *_= *x*_*ij*_*x*_*kl *_and obtain

(4) $$\max\sum_{(i,j)\in V_{1}\times V_{2}}\sigma (i,j)x_{ij}+\sum_{(i,j)\in V_{1}\times V_{2}}\sum_{(k,l)\in V_{1}\times V_{2}}\tau (i,j,k,l)y_{ijkl}$$

(5) $$\begin{array}{cc} \text{s}.\text{t}.\sum_{\left(i,j)\in \delta (v\right)}x_{ij}\leq 1 & \forall v\in V_{1}\cup V_{2} \end{array}$$

(6) *y*_*ijkl *_≤ *x*_*ij*_   ∀(*i*, *j*, *k*, *l*) ∈ (*V*_1 _× *V*_2_)^2^

(7) *y*_*ijkl *_≤ *x*_*kl*_   ∀(*i*, *j*, *k*, *l*) ∈ (*V*_1 _× *V*_2_)^2^

(8) *x*_*ij *_∈ {0, 1}   ∀(*i*, *j*) ∈ *V*_1 _× *V*_2_

(9) *y*_*ijkl *_∈ {0, 1}   ∀(*i*, *j*, *k*, *l*) ∈ (*V*_1 _× *V*_2_)^2^

We now apply *variable splitting *or *Lagrangian decomposition*, a well-known technique in mathematical programming [18], to build a good basis for a Lagrangian approach. In computational biology, this technique has already been successfully applied to the maximum contact map overlap problem in computational structural proteomics [19] and to structural RNA alignment [15].

We therefore split each structural variable *y*_*ijkl *_into two "directed" variables ${\vec{y}}_{ijkl}$ and ${\vec{y}}_{klij}$ and make sure that they adopt the same value. Likewise, we define new weights $\vec{\tau }$ for the directed structural variables with the property

(10) $$\vec{\tau }(i,j,k,l)+\vec{\tau }(k,l,i,j)=\tau (i,j,k,l),$$

setting $\vec{\tau }(i,j,k,l)=\vec{\tau }(k,l,i,j)=\frac{\tau (i,j,k,l)}{2}$. The resulting integer linear program is then:

(11) $$\max\sum_{(i,j)\in V_{1}\times V_{2}}\sigma (i,j)x_{ij}+\sum_{(i,j)\in V_{1}\times V_{2}}\sum_{(k,l)\in V_{1}\times V_{2}}\vec{\tau }(i,j,k,l){\vec{y}}_{ijkl}$$

(12) $$\begin{array}{cc} \text{s}.\text{t}.\sum_{\left(i,j)\in \delta (v\right)}x_{ij}\leq 1 & \forall v\in V_{1}\cup V_{2} \end{array}$$

(13) $$\begin{array}{cc} {\vec{y}}_{ijkl}\leq x_{ij} & \forall (i,j,k,l)\in {\left(V_{1}\times V_{2}\right)}^{2} \end{array}$$

(14) $$\begin{array}{cc} {\vec{y}}_{ijkl}={\vec{y}}_{klij} & \forall (i,j,k,l)\in {\left(V_{1}\times V_{2}\right)}^{2} \end{array}$$

(15) *x*_*ij *_∈ {0, 1}   ∀(*i*, *j*) ∈ *V*_1 _× *V*_2_

(16) $$\begin{array}{cc} {\vec{y}}_{ijkl}\in \{0,1\} & \forall (i,j,k,l)\in {\left(V_{1}\times V_{2}\right)}^{2} \end{array}$$

The following result allows us to concentrate on solving the ILP (11)–(16).

**Theorem 2. ***A feasible solution respecting the constraints of ILP (11)–(16) corresponds to a structural matching in the alignment graph whose score is equal to the score of the objective function*.

*Proof*. Let (*x*, $\vec{y}$) be a feasible solution of the ILP. Clearly, *x *represents a network alignment. Now consider a variable ${\vec{y}}_{ijkl}$ with ${\vec{y}}_{ijkl}$ = 1. Inequality (13) ensures that the first half of the match, namely, (*i*, *j*), is realized, whereas the second half is taken care of by equality (14) in combination with inequality (13). Thus, the solution corresponds to a structural matching, the score of which, due to property (10), clearly equals the score of (11). For the other direction of the proof, setting the variables *x *and *y *according to the characteristic vectors of a structural matching does not violate any of the constraints. Again, it is easy to see that the structural score of the matching and the objective function value coincide.   □

### Lagrangian relaxation for network alignment

Inspired by recent successes in solving similar integer linear programs using Lagrangian relaxation, we propose to employ this approach to find provably optimal and near-optimal solutions of ILP (11)–(16).

Therefore, we relax constraint (14) and obtain the following *relaxed problem*:

(17) $$\max\sum_{(i,j)\in V_{1}\times V_{2}}\sigma (i,j)x_{ij}+\sum_{(i,j)\in V_{1}\times V_{2}}\sum_{(k,l)\in V_{1}\times V_{2}}\vec{\tau }(i,j,k,l){\vec{y}}_{ijkl}+\sum_{(i,j)\in V_{1}\times V_{2}}\sum_{(k,l)\in V_{1}\times V_{2}}\lambda_{ijkl}({\vec{y}}_{ijkl}-{\vec{y}}_{klij})$$

(18) $$\begin{array}{cc} \text{s}.\text{t}.\sum_{\left(i,j)\in \delta (v\right)}x_{ij}\leq 1 & \forall v\in V_{1}\cup V_{2} \end{array}$$

(19) $$\begin{array}{cc} {\vec{y}}_{ijkl}\leq x_{ij} & \forall (i,j,k,l)\in {\left(V_{1}\times V_{2}\right)}^{2} \end{array}$$

(20) *x*_*ij *_∈ {0, 1}   ∀(*i*, *j*) ∈ *V*_1 _× *V*_2_

(21) $$\begin{array}{cc} {\vec{y}}_{ijkl}\in \{0,1\} & \forall (i,j,k,l)\in {\left(V_{1}\times V_{2}\right)}^{2} \end{array}$$

Here, vector *λ *contains the *Lagrangian multipliers*, which penalize the violation of (14). We exploit the fact that, in our case, *λ*_*ijkl *_= -*λ*_*klij *_and rewrite (17) as

(22) $$\max\sum_{(i,j)\in V_{1}\times V_{2}}\sigma (i,j)x_{ij}+\sum_{(i,j)\in V_{1}\times V_{2}}\sum_{(k,l)\in V_{1}\times V_{2}}(\vec{\tau }(i,j,k,l)+\lambda_{ijkl}){\vec{y}}_{ijkl}.$$

A fundamental result in mathematical optimization says that for each choice of penalty terms *λ*, each solution of the relaxed problem provides an upper bound for the original problem. Naturally, we are interested in the tightest such bound.

The penalty vectors in (22) change the weights of the structural matches and, intuitively, can be used to force pairs of complementary directed structural match variables to agree on their choices. We employ *subgradient optimization *for this task and find the Lagrangian multipliers that yield the lowest upper bound. Subgradient optimization is an iterative process that involves solving the relaxed problem over and over again, see, for example [20] for a detailed description of the method. The following result implies that we can do this efficiently.

**Theorem 3. ***The relaxed problem can be reduced to the bipartite matching problem and can be solved in polynomial time*.

*Proof*. The proof is similar to the one given in [19] for the contact map overlap problem and rests upon the observation that each directed structural match variable can be assigned unambiguously to an alignment variable – unlike in the undirected, original case. We can therefore concentrate on the alignment variables *x*. If such a variable *x*_*ij *_is zero, then its contribution to the objective function is zero as well, since all incident directed structural match variables ${\vec{y}}_{ijkl}$ are forced to zero due to constraint (19). If, on the other hand, an edge (*i*, *j*) is part of the solution, we can compute its contribution to the objective function, or its *profit*, in polynomial time as follows: we assign the weight $\vec{\tau }$(*i*, *j*, *k*, *l*) + *λ*_*ijkl *_to each edge (*l*, *m*) in the alignment graph and compute the profit *p*_*ij *_of edge (*i*, *j*) via a maximum bipartite matching according to these weights. The resulting matching corresponds to the best case that may happen if alignment edge (*i*, *j*) is part of the solution.

To compute the overall best solution, we choose those alignment edges that give the best network alignment according to their profits *p*. Again, this is a bipartite matching problem, which can be solved in polynomial time.   □

Theorem 3 gives us a good upper bound. In order to find good lower bounds, we analyze the network alignment given by the solution of each relaxed problem and compute the best *structural completion*, yielding a feasible solution for the original problem. Given a matching *M*, we simply add all structural matches (*i*, *j*), (*k*, *l*) with both (*i*, *j*) ∈ *M *and (*k*, *l*) ∈ *M*.

Let u*, l* be the best upper and lower bounds found by our algorithm, respectively, and let (x*, y*) be the best solution it finds. Our algorithm for network alignment is then as follows:

1 *Initialization*;

2 *λ *= 0; u* = ∞; l* = -∞;

3 *Main Loop*;

4 **repeat**

5   *x *= solution of relaxed problem with value *u*;

6   adapt Lagrangian multipliers;

7   compute structural completion (*x*, *y*) of *x *with value *l*;

8   **if ***u *<u* **then **u* = *u*;

9   **if ***l *> l* **then**

10      l* = *l*;

11      (x*, y*) = (*x*, *y*);

12 **until **l* = u* *or some termination criteria are met*;

As the structural matching problem is *NP*-hard, there will, in the general case, be a duality gap unless *P *equals *NP*. In other words, there will be instances for which u* and l* will not coincide. Therefore we define some additional termination criteria like, for example, a maximal number of iterations. Although the possible duality gap makes our algorithm heuristic in nature, it nevertheless comes with a quality guarantee due to the computation of the upper bound. Often this bound is quite good, and then it is fair to say that our algorithm efficiently computes provably near-optimal solutions. In addition, it is straightforward to embed the Lagrangian approach into a branch-and-bound framework resulting in a truly exact approach for the network alignment problem, which will then, of course, take exponential time to finish for some instances.

## Results and discussion

We have implemented that Lagrangian algorithm for network alignment described in the previous section and offer it as the freely available software tool NATALIE within the PLANET LISA framework. PLANET LISA is a library of algorithms for computational structural and systems biology, which has initially been created to facilitate computational structural comparisons of RNA molecules and proteins [21]. In its basic version, NATALIE reads in two graphs in GraphML format [22] as well as additional information that determine the *σ*- and *τ*-parts in the scoring function depending on the application.

The purpose of this paper is to introduce the new method; we have not yet performed a detailed comparative study including other tools, which we plan to carry out as future work. We present, however, preliminary results from two ongoing projects that utilize the NATALIE algorithm. These studies demonstrate that the method works well in practice and has a high potential to become a very competitive tool in the area of network alignment.

### First proof of concept: comparison of protein-protein interaction networks

In a cooperation with the Knowledge Management in Bioinformatics group of the Humboldt-Universität Berlin we use NATALIE to align protein-protein interaction networks based on orthology information about proteins in different species.

We analyze data from the following four species: *Homo sapiens*, *Mus musculus*, *Drosophila melanogaster*, and *Saccharomyces cerevisiae*. The PPI networks were obtained using data from several open databases and their origin is described in [11]. Candidates for orthologous proteins between the species were determined using protein enzyme classes, InterPro domains, and a minimum sequence identity threshold of *α *= 0.4, see again [11] for details. In a prototypical experiment, we compare the network of *H. sapiens *against all other networks using a simple scoring function. Table 1 provides information about the network sizes, where *n *and *m *denote the number of nodes and edges in the networks, and the average number of potential orthologs for a sequence identity threshold of *α *= 0.4 as compared to the network of *H. sapiens*. We use the following scoring function and align the three pairs of PPI networks. We set

$$\begin{array}{c} \sigma (i,j)=\{\begin{array}{ll} 0 & \text{if protein }i\text{ and }j\text{ are orthologous candidates}\\ -\infty & \text{otherwise} \end{array}\\ \tau (i,j,k,l)=\{\begin{array}{ll} 1 & \text{if both interactions }(i,k)\text{ and }(j,l)\text{ exist}\\ 0 & \text{otherwise}. \end{array} \end{array}$$

**Table 1:** Number of potential orthologs. Average number of potential orthologs as compared to *H. sapiens*.

Species	*n*	*m*	∅ cand. compared to *H. sapiens*
*H. sapiens*	9 695	34 979	n/a
*M. musculus*	3 247	3 116	5.47
*D. melanogaster*	10 232	41 332	2.87
*S. cerevisiae*	5 864	25 527	2.85

This scoring function simply counts the number of conserved interactions of proteins that are potentially orthologous. We limit the CPU time to 1 h and yield the results summarized in Tab. 2.

**Table 2:** Comparison of H. sapiens against other species. Results of comparing the PPI network of *H. sapiens *against other species. The entries in the table denote the instance, the value of the best solution found, the value of the upper bound, and the resulting quality guarantee.

*H. sapiens *vs.	best solution	upper bound	guarantee
*M. musculus*	1 087	1 087	100.00%
*D. melanogaster*	284	285	99.65%
*S. cerevisiae*	431	431	100.00%

**Figure 3:**
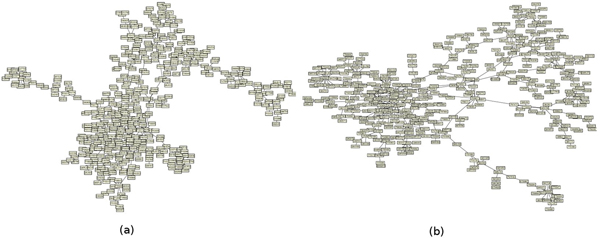
**Exemplary alignment of two larger PPI networks**. A maximum common protein-protein interaction network with respect to the number of conserved interactions in (a) *Homo sapiens *and (b) *Mus Musculus*.

Clearly, more elaborate scoring schemes may yield biologically more meaningful solutions. This simple experiment demonstrates, however, that the Lagrangian algorithm performs very well even on large data. All solutions but the alignment computed for *D. melanogaster *are provably optimal and even this alignment is very close to optimal. Figure 3 shows the alignment computed between the PPI networks of *Mus musculus *and *Homo sapiens*.

### Second proof of concept: classification of metabolic subnetworks

A common way to represent the topology of a metabolic network is its *stoichiometric matrix*, which characterizes the system of homogeneous linear equations that describe the network of biochemical reactions at steady state. Together with the Computational Systems Biochemistry group at Charité, Berlin, we investigate randomization models for a given metabolic network.

We therefore consider environments of different sizes for each reaction in the network and classify the resulting subnetworks according to their *topological equivalence*. Two reaction environments are topologically equivalent if the induced stoichiometric submatrices are permutation-equivalent, that is, one can be transformed into the other only by permuting its rows and columns. Then, randomized networks can be generated by swapping reaction environments that exhibit the same topology.

We employ a result by Colbourn [23] and determine permutation-equivalence via computing whether two corresponding labeled graphs are isomorphic. Since two graphs are isomorphic if and only if their maximum common subgraph equals the input graphs, we can use NATALIE to do the computations. We compute the equivalence classes of reaction environments of sizes *s *∈ {1, 2, 3} of the metabolic networks of *E. coli *and *S. cerevisiae*, which were obtained from the Systems Biology Research group at UCSD. The graphs that correspond to the stoichiometric matrices of these reaction environments are typically quite small and have rarely more than twenty vertices. Table 3 shows the number of pairwise comparisons that had to be computed:

**Table 3:** Classification of metabolic subnetworks. Number of comparisons for the classification of metabolic subnetworks depending on different reaction environment sizes *s*.

*s*	*E. coli*	*S. cerevisiae*
1	114 172	87 863
2	423 956	490 528
3	78 680 948	122 067 031

For each comparison, NATALIE has to decide whether the two subnetworks are topologically equivalent or not. Although, in the current version, it takes about two weeks to do the computations, NATALIE finds the correct answer for all of the more than 200 million comparisons and thus correctly computes the equivalence classes. In this application, the quality guarantee of the Lagrangian approach is indispensable, and the same results could not have been computed with a purely heuristic method. Yet, they could have been obtained probably much faster using a tailor-made algorithm for detecting graph isomorphisms. We plan, however, to develop a similarity metric between stoichiometric matrices based on the maximum common subgraph of their corresponding labeled graphs and have therefore used our novel approach, which has been proven efficient enough for this application. The details of this study will be described elsewhere.

## Conclusion

We believe that the maximum structural matching formulation and our algorithmic contribution is a first step towards a very competitive framework for network alignment, query, and comparison problems. We see perspectives for many interesting research directions. As the formulation as well as the algorithm can deal with multiple alignments, we plan to adapt the concepts to the multiple case. For practical purposes, a progressive alignment method seems to be appropriate for which an adequate consensus concept has to be developed. Moreover, the analogy to classical sequence alignment suggests to investigate *local network alignments*, where a first step consists in computing maximum *connected *motifs.

As the formulation is very flexible, it can easily be adapted to any type of undirected or directed, labeled or unlabeled, and weighted or unweighted network. It can be used for answering network queries as well as for detecting repeated motifs in a single network.

Clearly, a good search procedure is only one component in a successful alignment framework. The analogy to sequence alignment suggests that a lot of further research has to go into development and evaluation of suitable scoring functions and into statistical analysis of the results. This more statistically-oriented line of research will be different for each of the numerous applications for network alignment in computational biology. Currently, we address these topics in the ongoing projects, the alignment of PPI networks and the comparison of metabolic networks. Likewise, a visualization of the results is an important research topic. Here, we envision an integration into the CYTOSCAPE software [24].

## Competing interests

The author declares that they have no competing interests.
